# Supplementary material for: qTorch: The quantum tensor contraction handler
Source: PLoS One. 2018 Dec 10;13(12):e0208510. doi: 10.1371/journal.pone.0208510 (PMC6287880; doi:10.1371/journal.pone.0208510)
Supplement: S1 Appendix — (PDF) [file pone.0208510.s001.pdf]

S1 Appendix for “qTorch: The quantum tensor contraction handler”

E. Schuyler Fried[1†], Nicolas P. D. Sawaya[1,2†], Yudong Cao[1], Ian D. Kivlichan[1], Jhonathan Romero[1], Alán Aspuru-Guzik[1,3*]

1 Department of Chemistry and Chemical Biology, Harvard University, Cambridge, MA 02138, USA
2 Intel Labs, Intel Corporation, Santa Clara, CA 95054, USA
3 Canadian Institute for Advanced Research, Toronto, Ontario M5G 1Z8, Canada

†These authors contributed equally to this work.
* alan@aspuru.com

Descriptions of Algorithms

QAOA

QAOA attempts to approximate solutions for satisfaction problems, in which one attempts to satisfy many clauses at once. The accuracy depends upon a parameter p—increasing p results in a better approximation. In a more general sense, combinatorial optimization problems can be defined by an objective function with a variable number of binary clauses and bits per clause. When finding approximate solutions, the goal is to maximize or minimize the number of clauses satisfied. This class of objective functions is defined by the sum of all of its n clauses:

$$C(z) = \sum_{k=1}^{n} C_k(z) \tag{1}$$

where z, a binary string of fixed length, is the input to optimize. Each clause $C_k(z)$, if satisfied, outputs 1 and if not, outputs 0.

We create an operator $U(C, \gamma)$, which is defined as

$$U(C, \gamma) = e^{-i\gamma C} = \prod_{k=1}^{n} e^{-i\gamma C_k}. \tag{2}$$

Note that each operator in the product $e^{-i\gamma C_k}$ is local to the qubits acted on by the clause C_k. Additionally, all operators in the sum commute because all are diagonal.

Next, create a second operator $U(B, \beta)$. The operator does not depend on the objective function (while $U(C, \gamma)$ does) and is defined on q qubits by

$$U(B, \beta) = e^{-i\beta B} = \prod_{i=1}^{q} e^{-i\beta \sigma_i^x}. \tag{3}$$

These are the only two operators used in QAOA, applied to the starting state $|s\rangle = |+\rangle^{\otimes q}$, where $|+\rangle = \frac{|0\rangle + |1\rangle}{\sqrt{2}}$.

The parameter p determines how many times the U_C and U_B operators are applied to the initial state. If $p = 1$, there is only one γ and one β, and U_C and U_B are only applied once:

$$U(B, \beta)U(C, \gamma) |s\rangle = |\gamma, \beta\rangle \tag{4}$$

If p is greater than 1, there are p number of γ angles and p number of β angles, so that $2p$ operators are applied to the state. The resulting state for parameter p is

$$U(B, \beta_p)U(C, \gamma_p) \cdots U(B, \beta)U(C, \gamma) |s\rangle = |\gamma, \beta\rangle \tag{5}$$

Max-Cut

Definition

Max-Cut is a prototypical optimization problem in graph theory. The input is a graph represented by a vector of edges, where each edge connects two vertices in the graph. The solution is the binary string z where each bit corresponds to a vertex, and z maximizes the number of cut edges. A “cut” edge means that the two vertices connected by the edge have opposite values.

To approximate a solution to the Max-Cut problem using QAOA, one represents each vertex in the graph by one qubit. The objective function for Max-Cut is

$$C = \sum_{\langle ij \rangle} C_{\langle ij \rangle}, \tag{6}$$

where

$$C_{\langle ij \rangle} = \frac{1}{2}(1 - \sigma_i^z \sigma_j^z), \tag{7}$$

and each edge is represented by i and j, the qubit indices of the two vertices it connects.

Max-Cut on 3-Regular Graphs

It is notable that the parameter p can be interpreted as determining how far the algorithm “sees” when it maximizes the value of each of the objective function clauses (Farhi et al. 2014, arXiv:1411.4028). In the case of a 3-regular graph, the i and j vertices can only have a maximum of two other vertices each that they are connected to. Therefore, the maximum number of qubits involved in computing the cost of each objective function’s clause is six. Below is a subgraph that illustrates the “locality” of the algorithm when $p = 1$:

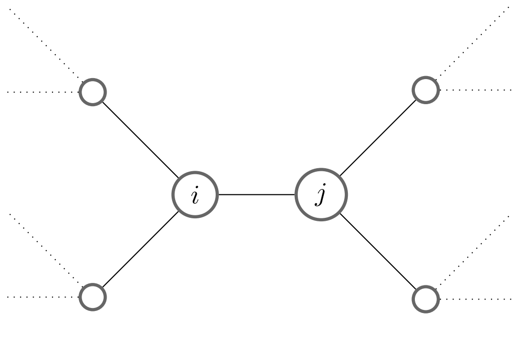

For $p = 2$, to evaluate the cost of each clause in the objective function, the algorithm “sees” every vertex within a distance two of i and j. Hence the maximum number of qubits that are involved in calculating the cost of one clause is 14:

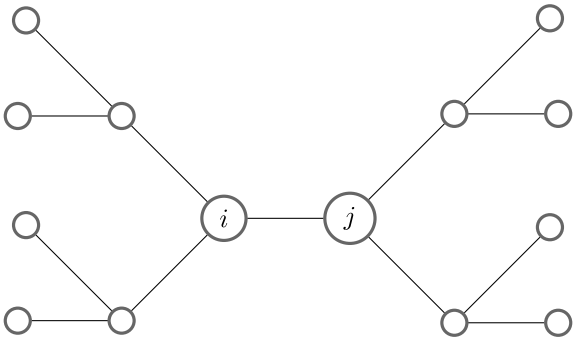

LIQUi| > benchmark

The quantum circuit simulations performed on LIQUi| > are used as benchmarks for comparing with our qTorch implementation. First, a `.qasm` file describing the quantum circuit is converted to an F# script containing a function that runs the circuit in LIQUi| >. When the circuit has many gates (say, with more than 500 gates), multiple functions are constructed in the F# script, each containing a subset (say, 500 gates) of the quantum circuit. We then use LIQUi| > to first compile the functions(s) corresponding to (subsets of) the circuit, then run the circuit and finally compute the expectation value $\langle M \rangle$ of some user-specified operator M with respect to the final state of the circuit. We compute the total wall clock time of the three-step process and use it as a comparison to our qTorch performance.

Here are a few additional notes concerning the LIQUi| > benchmark:

1. For computing the expectation value $\langle M \rangle$, we assume that M is a string of Pauli operators *i.e.* operators of the form $P_1 \otimes P_2 \otimes \cdots \otimes P_n$ where each $P_i \in \{X, Y, Z, I\}$ is a Pauli operator. Our method for computing $\langle M \rangle$ takes advantage of this property and runs in time that scales linearly as the number of non-zero amplitudes in the final state. Timing results from the benchmark circuits indicate that the overhead for computing $\langle M \rangle$ is only a small fraction (at most 10%) of the time spent running the circuit.

2. Some of the quantum circuits that we use for benchmarking are circuits for quantum chemistry simulations. Although LIQUi| > has a more optimized implementation specifically dedicated for quantum chemistry simulation, we choose not to take advantage of such implementation because our focus is on the average performance of simulating general quantum circuits.

3. Our LIQUi| > benchmark is performed using a Docker container to ensure that one could execute the programs regardless of the operating system used. The environment inside the container is Unix-like and `mono` is used for running Windows `.exe` executables. Due to the upper limit on the stack size imposed by `mono`, we restrict each function in our F# script generated from the `.qasm` file to have at most 500 gates, and we use LIQUi| > to compile each function individually. This partition of the quantum circuit into segments of at most 500 gates renders the simulation speed possibly sub-optimal compared with the case where LIQUi| > is used for compiling the entire quantum circuit in one shot. However, the circuits that we simulate have been partitioned into no more than 10 sub-functions, thus we believe that our implementation should at least capture the order of magnitude of the optimized speed of LIQUi| > simulation.
